# 186. The Role of Repeated Blood Cultures in Hematologic Malignancy Patients with Neutropenic Fever due to Gram-Negative Bacterial Bloodstream Infections

**DOI:** 10.1093/ofid/ofad500.259

**Published:** 2023-11-27

**Authors:** Nitya Dhanaraj, Carlo Foppiano Palacios

**Affiliations:** Montefiore Medical Center/AECOM, Branchburg, New Jersey; Cooper University Health Care, Camden, New Jersey

## Abstract

**Background:**

Several observational studies and a recent meta-analysis suggest there is a mortality benefit associated with acquiring repeat blood cultures for gram-negative bacterial bloodstream infections (GNBSI). However, limited data are available among patients with febrile neutropenia. We sought to evaluate the utility of obtaining follow-up blood cultures (FUBC) in GNBSI among neutropenic hematological malignancy patients.

**Methods:**

A retrospective chart review was conducted to identify all patients with a hematologic malignancy admitted with a diagnosis of neutropenic fever due to gram-negative rod bacteremia (GNRB) from 2018-2021 at a large urban academic medical center. We collected demographics, cancer diagnosis and treatment data, microbiology and antibiotic information, and death at discharge, 30 days, and 90 days. Descriptive statistics and chi-square tests were used.

**Results:**

A total of 47 episodes of GNBSI among 43 patients were included. Mean age was 57 years, 61% were male, 49% were White, and 14% were Latino. Most patients had AML (47%), and most common chemotherapy regimen was R-CHOP (19%). 32% of GNRB were due to *K. pneumoniae*, in the setting of long-term central venous catheter (53%), and from a gastrointestinal source (49%). FUBC were collected among most patients (87%), but only two patients (5%) had positive FUBC. ESBL resistance profiles were uncommon (6%), and most were treated with beta-lactams (55%). Patients who died at discharge were less likely to have FUBC collected (p< 0.001). We also found an association between acquiring FUBC and mortality at 30 (p=0.01) and 90 days (p=0.02). However, there was no association between having a positive FUBC and mortality at discharge (p=0.47), 30 days (p=0.93), and 90 days (p=1.00).

**Conclusion:**

Among patients with neutropenic fever found to have GNBSI, acquiring repeat blood cultures was associated with decreased mortality. Due to their immunocompromised status, patients with neutropenic fever and GNRB should have repeat blood cultures collected.

**Disclosures:**

**All Authors**: No reported disclosures

